# Oil of Eucalyptus to Cure Chilblains

**Published:** 1898-05

**Authors:** 


Oil of eucalyptus, frequently applied with a camel's-hair pencil over the surface of chilblains, relieves pain and soon cures them.—*Med. Summary.*

